# Case Report: Glofitamab in the treatment of a patient with central nervous system-involved Burkitt lymphoma

**DOI:** 10.3389/fonc.2026.1724213

**Published:** 2026-02-17

**Authors:** Yuejiao Huang, Jiawen Jiang, Jinfeng Lu, Chunfeng Sun, Juan Qian, Xuefen You, Zenghua Lin

**Affiliations:** 1 Department of Hematology, Affiliated Hospital of Nantong University, Nantong, Jiangsu, China; 2 Department of Hematology, Medical School of Nantong University, Nantong, Jiangsu, China; 3 Department of Nuclear Medicine, Affiliated Hospital of Nantong University, Nantong, Jiangsu, China

**Keywords:** Burkitt lymphoma (BL), central nervous system (CNS), complete metabolic response (CMR), glofitamab, relapsed/refractory

## Abstract

Burkitt lymphoma (BL) is a rare and highly aggressive B-cell non-Hodgkin lymphoma. While targeted combination chemotherapy can be effective, it is associated with a high rate of relapsed or refractory disease and a pronounced propensity for central nervous system (CNS) involvement. Currently, novel therapies such as chimeric antigen receptor T-cell (CAR-T) therapy have not demonstrated established efficacy in BL. Given the poor prognosis and the challenge of managing relapsed/refractory BL, the use of bispecific antibodies, specifically glofitamab, as employed in this case, has yielded a favorable therapeutic outcome. This is particularly noteworthy in the present case, given the patient’s documented CNS infiltration. After the failure of first-line treatment in this case, the combination of glofitamab and a Bruton’s tyrosine kinase inhibitor (BTKi) was used for six cycles, which significantly improved the patient’s CNS infiltration and achieved a long remission period. This provides an opportunity to try glofitamab in the treatment of CNS lymphoma, and we look forward to its confirmation in more BL patients.

## Introduction

Burkitt lymphoma (BL) is a highly aggressive malignant neoplasm that can occur in both children and adults. BL can occur at any age and demonstrates a male predominance. Endemic BL primarily affects children, whereas the sporadic and immunodeficiency-associated subtypes are more frequently observed in adults, with incidence peaks around age 50 and in the elderly. US epidemiological data indicate a trimodal age distribution for male BL patients, with peaks at approximately 10, 75, and a third peak at 40 years of age (1). Sporadic BL often manifests with abdominal involvement, particularly affecting the gastrointestinal tract, retroperitoneum, liver, and kidneys. It may also present as a leukemic phase disease, with 30-35% of patients exhibiting bone marrow infiltration. Immunodeficiency-associated BL shows a higher propensity for lymph node and CNS involvement compared to other subtypes (2). It is characterized by dysregulation of the c-MYC gene, present in approximately 80% of all cases, with the t(8;14)(q24;q32) translocation being the most frequent genetic hallmark (3). Alterations in the c-MYC gene drive tumorigenesis by promoting uncontrolled cell proliferation, evading apoptotic regulation, and ultimately impacting treatment efficacy (4). In BL, c-MYC dysregulation leads to sustained tumor cell proliferation and activates B-cell receptor signaling, which interacts with the PI3K pathway to enhance cell survival (5). The Epstein-Barr virus (EBV) infection status is also a significant factor influencing clinical risk, as different EBV statuses reflect distinct molecular profiles and pathogenic mechanisms (6). Consequently, moving away from the previous classification into endemic, sporadic, and immunodeficiency-associated subtypes, the current WHO-HAEM 5 recommends subtyping BL based on EBV status: EBV-positive and EBV-negative (7). The pathogenesis of BL is widely studied; a prevailing hypothesis suggests that viral infection of nodal B-cells precedes MYC translocation, subsequently triggering lymphomagenesis. However, alternative data challenge this “virus-first” hypothesis and propose a “MYC-first” model, wherein MYC translocation is the initial event, and EBV’s anti-apoptotic effects act synergistically to favor lymphomagenesis.

Central nervous system (CNS) involvement is observed in 30% to 50% of cases, posing additional challenges for disease control (2). Two large-scale international clinical studies found that patients under 40 years of age, with a performance status <2, serum lactate dehydrogenase level <3 times the upper limit of normal, and without CNS involvement had a 3-year overall survival rate of 96%; however, this group constituted only 18% of the cases. In contrast, patients with ≥2 risk factors (46% of cases) had an expected long-term survival probability of only 59%. The prognosis is significantly worse for elderly patients, with survival rates below 10%, likely attributable to their intolerance to intensive treatment regimens and the presence of inherent disease risk factors (8, 9).

## Case description

**Figure 1:**
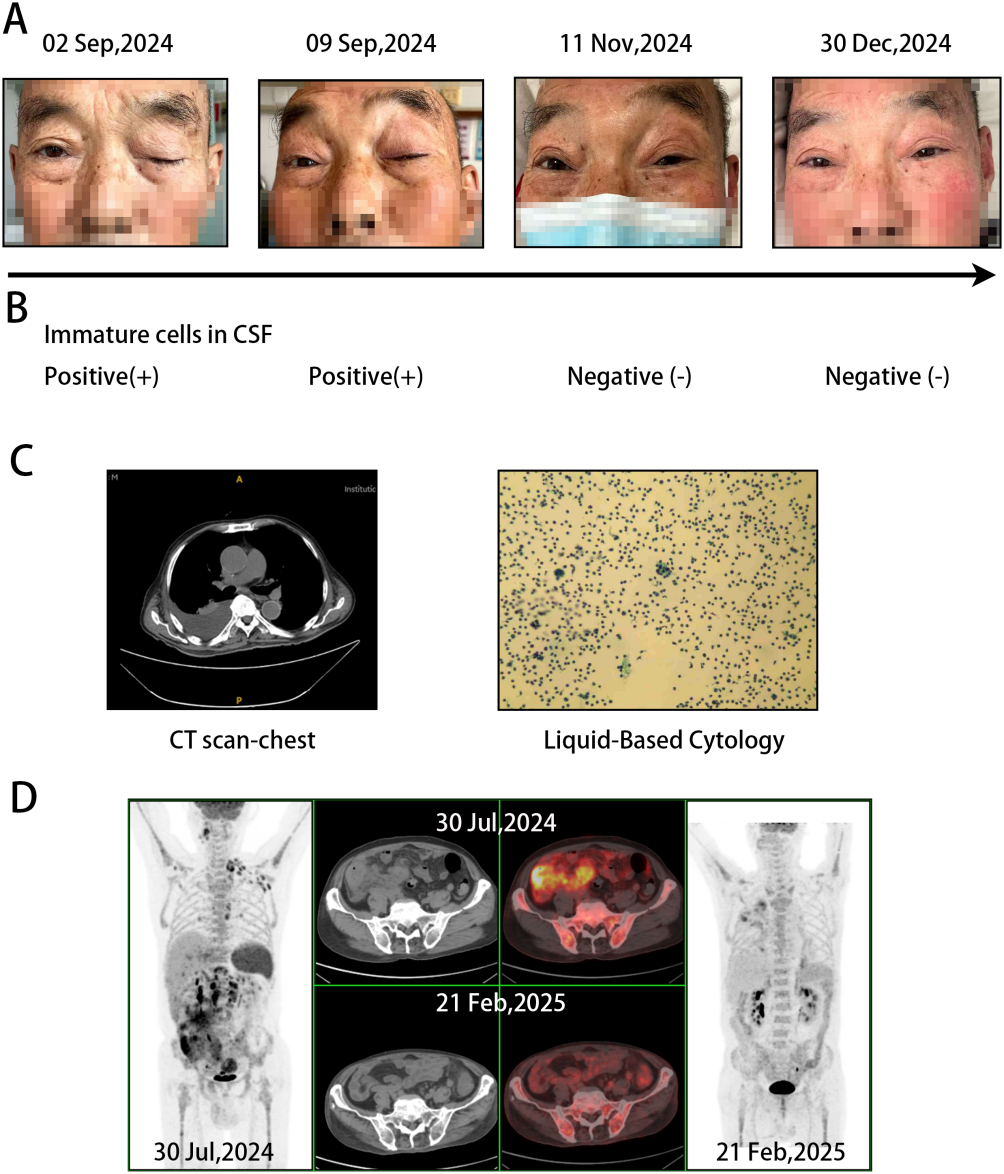
Therapeutic outcomes. **(A)** Resolution of ptosis symptoms in the patient. **(B)** Clearance of malignant cells in the CSF. **(C)** CT images of pleural effusion and corresponding cytological findings during treatment. **(D)** PET-CT scan showing changes in lesion metabolic activity, indicating a CMR.

**Figure 2:**
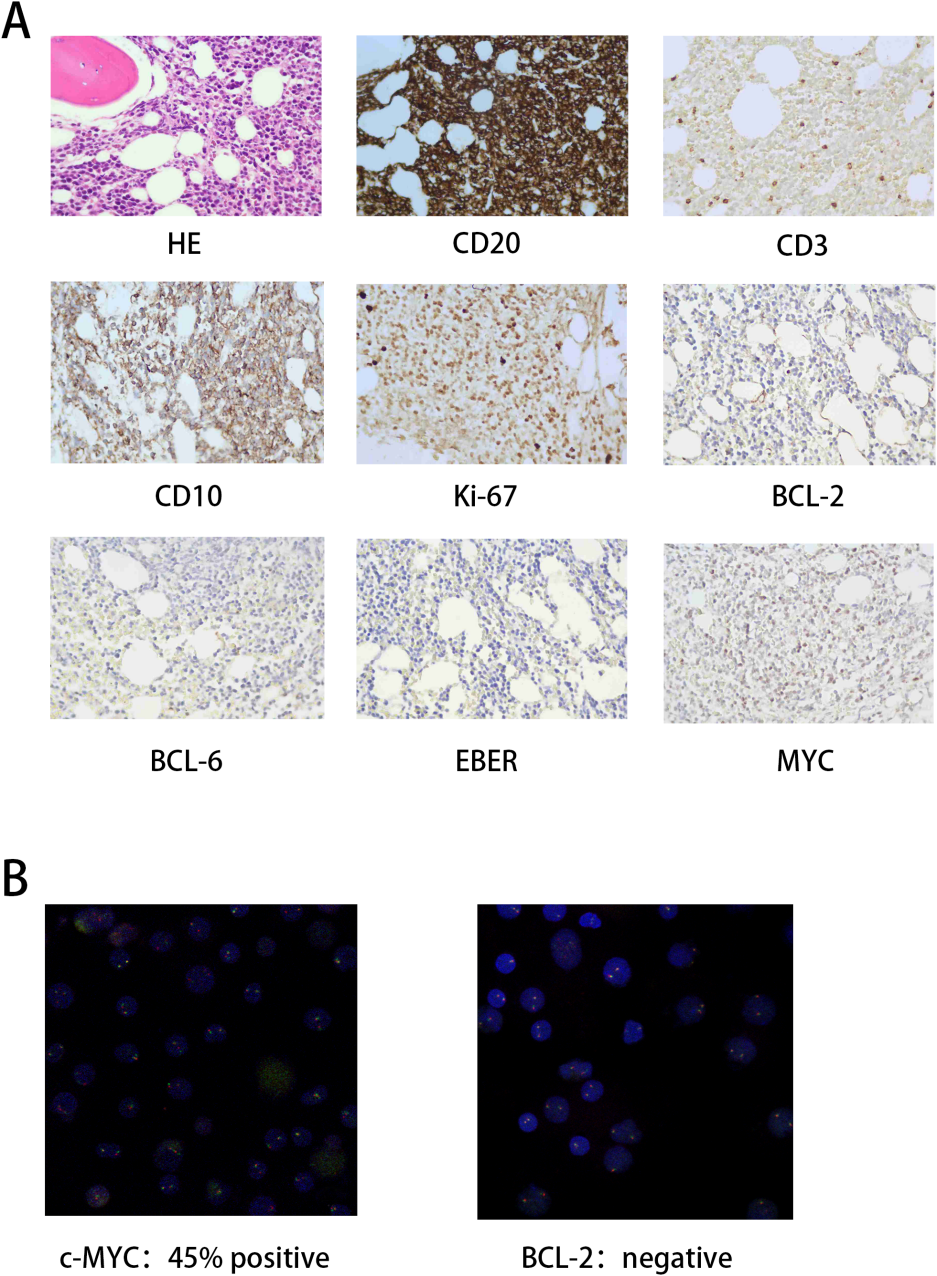
Pathological diagnostic findings. **(A)** H&E staining results and immunohistochemistry (IHC) expression profiles. **(B)** Fluorescence *in situ* hybridization (FISH) indicates c-MYC and BCL-2 expression.

The patient is a 78-year-old elderly male who was admitted to the hospital due to abdominal pain. A CT scan suggested thickening of the ileal wall with multiple enlarged and partially confluent lymph nodes in the abdominal cavity and retroperitoneum, raising suspicion for lymphoma. A subsequent positron emission tomography-computed tomography (PET-CT) scan revealed systemic lymphadenopathy with increased metabolic activity, suggestive of possible hepatic, splenic, and bone marrow involvement (**Figure 1D** left, SUVmax: 14.2). Bone marrow biopsy pathology confirmed BL. The immunophenotype was: CD19(+), CD20(+), CD10(+), CD138(partial+), CD21(-), CD5(-), CD3(T-cells+), Mum-1(-), BCL-2(-), BCL-6(-), Ki-67(60%+) (**Figure 2A**). FISH analysis was positive for c-MYC rearrangement (45% of cells) and negative for BCL-2 (**Figure 2B**). The tumor was EBV-negative. A comprehensive diagnosis of Stage IV BL was established. At initial diagnosis, the patient was assessed as having a high-risk profile. The international prognostic index (IPI) score was 5, based on stage IV disease, age >60 years, involvement of two or more extranodal sites, an eastern cooperative oncology group (ECOG) performance status of 2, and a markedly elevated serum lactate dehydrogenase (LDH) level of 3780 U/L. Serology testing confirmed a negative HIV status. The absolute lymphocyte count (ALC) was 1.26 × 10^9^/L.

First-line therapy consisted of R-CHOP like (ECOG score: 2, body surface area 1.72 m²; Cyclophosphamide 0.6g d1,4 + Hydroxydaunorubicin 20mg d1 + Vincristine 2mg d1 + Prednisone 40mg d1-5+ Rituximab 600mg d0). The patient refused further lumbar puncture and preventive intrathecal injection. After a follow-up bone marrow puncture smear examination indicated remission, the patient was discharged. Prior to the second treatment cycle, the patient experienced a sudden onset of unilateral ptosis (**Figure 1A**, left). Lumbar puncture revealed the presence of blast cells in the cerebrospinal fluid (**Figure 1B**, left), confirming CNS involvement. This was accompanied by a significant right-sided pleural effusion (**Figure 1C**, left); cytological examination of the pleural fluid identified a proliferation of lymphoid cells (**Figure 1C**, right).

Second-line therapy was initiated with Glofitamab 30mg + Zanubrutinib 160mg twice daily. Obinutuzumab 1000mg was administered as pre-treatment before the first cycle of Glofitamab, followed by the Glofitamab dose-escalation protocol. Following the first infusion of glofitamab, the patient developed a fever with a peak temperature of 38.7 °C on day 1, which was accompanied by hypoxia requiring low-flow oxygen supplementation (2 L/min). This clinical presentation was consistent with grade 2 cytokine release syndrome (CRS) according to ASTCT criteria. The patient was promptly treated with a single dose of tocilizumab (480 mg) and supportive care, resulting in the rapid resolution of symptoms. No subsequent episodes of CRS were observed. Importantly, the patient did not exhibit any symptoms suggestive of immune effector cell-associated neurotoxicity syndrome (ICANS) during the entire treatment period. Concurrent intrathecal chemotherapy with Methotrexate 12mg was administered during treatment, which was synchronized with the systemic Glofitamab treatment cycles. To achieve rapid cytoreduction and symptomatic control during the initial phase, an intensified frequency of once to twice per week was utilized.

The patient’s condition gradually improved, with an increasing ability to open the affected eye. After 6 cycles of therapy, the ptosis had resolved completely (**Figure 1A**, right). Cerebrospinal fluid (CSF) examinations were performed during each treatment cycle. Consistent with the clinical course, immature cells were initially detected in the CSF in early September 2024 (**Figure 1B**, left). This was followed by the first conversion to a negative result in November 2024, which has been sustained to date (**Figure 1B**, right). At initial presentation, imaging revealed marked circumferential thickening of the ileal wall (SUVmax 12.7) with perienteric exudate, extensive lymphadenopathy in multiple regions (largest 2.2 cm, SUVmax 14.2), hepatosplenomegaly, and diffusely increased bone marrow fluoro deoxy glucose (FDG) uptake. Following six cycles of therapy, all previously noted lesions demonstrated significant reduction in both size and metabolic activity, achieving a Deauville score of 3 and indicating a complete metabolic response (CMR) (**Figure 1D**). Concurrently, scattered inflammatory lesions were also observed in the right lung, which subsequently resolved with active antibiotics treatment, along with the disappearance of the pleural effusion. The patient did not enter maintenance treatment after six cycles of treatment due to the good therapeutic effect and the high cost of the drug, glofitamab. The patient is currently maintained on oral Zanubrutinib therapy and is undergoing regular follow-up in the outpatient clinic, with no obvious recurrence to date (04 Nov. 2025). Specific treatment dates and dosages are detailed in **Table 1**.

**Table 1:** Treatment timeline for the patient.

Date	Treatment regimen (BSA: 1.72 m²)	Efficacy assessment	Remarks/Notes
24-08-04	Rituximab 600mg		
24-08-05	Cyclophosphamide 0.6g, Hydroxydaunorubicin 20mg Vincristine 2mg, Prednisone 40mg		Prednisone 40mg continued until 24-08-10
24-08-09	Cyclophosphamide 0.6g, Prednisone 40mg		
24-09-02、09-05、09-10、09-14	Intrathecal injection: Cytarabine + Methotrexate	Progressive Disease	Onset of ptosis
24-09-04	Obinutuzumab 1000mg,		
24-09-11、	Glofitamab 2.5mg		Grade 2 CRS
24-09-25	Glofitamab 10mg		
24-10-14			Large pleural effusion, catheter drainage
24-10-21	Glofitamab 30mg + Intrathecal Injection: Cytarabine	Partial Response	
24-11-12、12-04、12-31	Glofitamab 30mg+ Zanubrutinib 160mg bid		Pulmonary abscess, anti-infective therapy
25-01-07	Intrathecal Injection: Cytarabine		
25-01-26	Glofitamab 30mg+ Zanubrutinib 160mg bid		Initiated prophylactic anti-TB therapy (25-01-10)
25-02-21		Complete Metabolic Response	

## Discussion

The clinical presentation of BL is typically aggressive and rapid in onset, characterized by elevated lactate dehydrogenase levels above the upper limit of normal, involvement of the colorectum (especially the ileocecal region), large tumor masses (≥7 cm), particularly in the abdomen, and advanced disease stage (III or IV). These features, along with rapid disease progression, warrant heightened clinical vigilance (10). The present case involves an elderly male patient with EBV-negative but c-MYC-altered BL. His initial presentation was dominated by abdominal symptoms with bone marrow involvement, followed by CNS infiltration and thoracic involvement during the treatment course. The pathological diagnosis of this case was reached through a joint discussion by multiple experts in the pathology department, in combination with bone marrow flow cytometry. The reason why the patient’s Ki-67 index was lower than that of a typical BL was considered to be due to the fact that the biopsy was taken from the bone marrow, and the decalcification process of the specimen to some extent affected its expression. Combined with the immunophenotypic profile, these findings are consistent with sporadic BL, indicating a poor prognosis. Moreover, the rapid progression of the patient’s condition did not fully align with the Ki-67 index. The patient’s predilection sites and central nervous system involvement were more consistent with sporadic BL. At that time, the patient’s condition was severe, and treatment was initiated immediately after the bone marrow biopsy, leaving no time for us to obtain a pathological diagnosis of the abdominal lesion, which was a regrettable aspect of this case.

Patients with BL experiencing CNS relapse face extremely poor outcomes, with a median overall survival of just 2.8 months. This underscores the clinical imperative for incorporating CNS prophylaxis into treatment strategies for all high-risk patients (11). Rituximab-based regimens, such as R-CHOP or DA-EPOCH-R, are not recommended for patients with confirmed CNS involvement at baseline, primarily due to the lack of agents within these regimens that adequately penetrate the blood-brain barrier. This elderly patient, though presenting with highly aggressive disease, was frail and intolerant of high-intensity chemotherapy. The initial choice of R-miniCHOP resulted in suboptimal disease control and rapid progression, potentially attributable to the regimen’s inability to cross the blood-brain barrier. As there was no evidence of CNS infiltration at baseline, prophylactic intrathecal therapy was not administered, likely contributing to the swift CNS progression. However, it is noteworthy that 15-25% of patients with CNS lymphoma do not respond to high-dose methotrexate chemotherapy, and 25-50% of those who initially respond experience relapse, with higher recurrence rates observed in elderly patients (12). When considering salvage therapy options combining intrathecal MTX with subsequent systemic treatment, a deliberation between chimeric antigen receptor T-cell (CAR-T) cell therapy and bispecific antibodies ensued. Although CAR-T cell therapy represents a potent treatment option for many patients with relapsed or refractory B-cell lymphomas after one or more prior lines of therapy, its application in this case was weighed against factors including the patient’s baseline condition, disease characteristics, previous treatments, and the heterogeneity, toxicities, and logistical challenges associated with CAR-T product delivery. Furthermore, the reported median follow-up for CAR-T therapy in such contexts is relatively short (approximately 4.7 months), indicating current limitations (13). Consequently, glofitamab was selected as the subsequent therapy, yielding unexpectedly remarkable efficacy.

Glofitamab is a commercially available, off-the-shelf T-cell-engaging bispecific antibody featuring a 2:1 configuration with bivalent binding to CD20 on B cells and monovalently binding to CD3 on T cells (14). In transplant-ineligible patients with relapsed or refractory B-cell lymphomas, particularly in the more extensively studied context of diffuse large B-cell lymphoma, data from the phase III STARGLO trial demonstrated that glofitamab combined with chemotherapy yielded significantly superior overall survival, progression-free survival, and objective response rate compared to a rituximab plus chemotherapy regimen (15, 16). These findings have been further corroborated in real-world settings (17). Previous investigations have revealed that although the mean concentration of glofitamab in the cerebrospinal fluid is only 0.1-0.4% of that in peripheral blood, this low concentration was sufficient to safely alleviate symptoms in patients with secondary CNS lymphoma, which is superior to traditional CD20 monoclonal antibodies (18). The 2022 Chinese expert consensus on the management of primary CNS lymphoma recommended that the Bruton’s Tyrosine Kinase (BTK) inhibitor, either in combination with or without high-dose chemotherapy, could be used as a re-induction regimen for refractory/relapsed primary CNS lymphoma patients. BTK inhibitors have a relatively high cerebrospinal fluid/plasma ratio in patients, which can exceed 25% (19). Zanubrutinib, a second-generation BTK inhibitor, has demonstrated favorable safety and efficacy profiles in the treatment of CNS lymphoma according to a retrospective study, along with excellent blood-brain barrier penetration (20).

Our case highlights the exceptionally prominent performance of glofitamab in treating CNS-involved BL, achieving a clinical breakthrough characterized by the significant resolution of multiple lesions, gradual disappearance of clinical symptoms, and a remarkable therapeutic outcome. Regarding future prospects, consolidation with transplantation may offer higher response rates. Nevertheless, given this patient’s advanced age, he was not a suitable candidate for subsequent transplantation. He is currently under active clinical follow-up, and we anticipate a prolonged clinical remission period. Should disease progression occur later, the feasibility of re-challenging with glofitamab warrants further clinical exploration and represents a potential area for future breakthrough. Unfortunately, in this case, we were unable to measure the blood drug concentrations of the monotherapy of glofitamab and its combination with BTK inhibitors, nor could we assess whether they act together or have a synergistic effect. There was no further research on the combined intrathecal injection. Due to the low incidence, this study has no more cases to support the role of glofitamab in BL with CNS infiltration. Although the patient responded well to the treatment, there was no further maintenance treatment, and the follow-up period was less than 12 months, lacking long-term follow-up data. The International BL Network (www.burkitt-lymphoma.org) is currently developing a global registry aimed at facilitating clinical trials, translational research, and large-scale data analysis for BL. Nonetheless, advancing the global diagnosis and treatment of BL still necessitates substantial financial and pharmaceutical research and development support from governments and the healthcare industry. We look forward to witnessing substantial and rapid improvements in the management of BL in the future.

## Data Availability

The original contributions presented in the study are included in the article/supplementary material. Further inquiries can be directed to the corresponding author.
